# Ophthalmic Artery Doppler at 11–13 Weeks’ Gestation and Birth of Small-for-Gestational-Age Neonates

**DOI:** 10.3390/jcm14134425

**Published:** 2025-06-21

**Authors:** Nicoleta Gana, Dragana Ianosev, Nima Allafi, Mechmet Impis Oglou, Kypros H. Nicolaides

**Affiliations:** 1 Faculty of Medicine, Carol Davila University of Medicine and Pharmacy, 020021 Bucharest, Romania; gana_nicoleta@yahoo.com; 2 Harris Birthright Research Centre for Fetal Medicine, King’s College, London SE5 8BB, UK; dragana.ianosev@nhs.net (D.I.); nima.allafi@nhs.net (N.A.); mechmet.impisoglou@nhs.net (M.I.O.)

**Keywords:** small for gestational age, ophthalmic artery Doppler, first trimester

## Abstract

**Background/Objective:** Small-for-gestational-age (SGA) status constitutes a significant risk factor for adverse neonatal outcomes and predisposes individuals to long-term health complications. Detecting pregnancies at risk early in gestation could significantly improve perinatal outcomes. Recent evidence suggests that ophthalmic artery Doppler assessment in the first trimester may contribute to the prediction of impaired placentation reflected in increased risk for preeclampsia. This study aimed to investigate the association between first-trimester ophthalmic artery Doppler parameters and the subsequent birth of small-for-gestational-age (SGA) neonates. **Methods:** In this prospective observational analysis, 4054 pregnant women underwent ophthalmic artery Doppler evaluation at 11–13 weeks gestation. Maternal demographics, biophysical and biochemical markers, and ophthalmic artery Doppler measurements of pulsatility index (PI) and peak systolic velocity (PSV) ratio were obtained. Outcomes were classified based on birthweight into the ≤3rd percentile and >3rd percentile and ≤10th percentile and >10th percentile groups. To determine the predictive value of Doppler indices, statistical methods included comparative analyses and the receiver operating characteristic (ROC) curves. **Results:** The analysis indicated that increased PSV ratio at 11–13 weeks gestation correlated with an increased risk of SGA. The PI was not found to be a significant discriminator between pregnancies complicated by SGA and non-SGA pregnancies. **Conclusions:** First-trimester ophthalmic artery Doppler assessment offers promise as a non-invasive technique for the early identification of pregnancies at risk for SGA neonates. Further validation through large, multicenter studies is needed to confirm its utility and to standardize its use in clinical protocols.

## 1. Introduction

Ophthalmic artery Doppler assesses the blood flow in the ophthalmic artery, a branch of the internal carotid artery, characterized by a two-peak systolic waveform. The first peak (PSV1) reflects cardiac output, while the second (PSV2) is influenced by peripheral vascular resistance. The ratio between these peaks (PSV ratio) serves as an indicator of systemic vascular resistance and arterial compliance [1,2,3]. Given the shared pathophysiological mechanisms between hypertensive disorders and birth of small-for-gestational-age (SGA) neonates—such as impaired placentation and endothelial dysfunction—ophthalmic artery Doppler may provide an early, non-invasive marker for identifying pregnancies at risk of SGA, complementing traditional uterine artery assessments [4,5].

Fetal growth restriction and preeclampsia are characterized by impaired placental function, endothelial dysfunction, and abnormal adaptation of the mother to the pregnancy [6]. These pathologies are more likely to reoccur in further pregnancies [7].

The ophthalmic artery Doppler has already been studied and proven as a screening tool for preeclampsia. Ophthalmic artery Doppler indices could be used from the first trimester and throughout the pregnancy to predict early and late preeclampsia [8,9].

SGA refers to a situation where a newborn weight or abdominal circumference is below the 10th percentile for its own gestational age. SGA is a significant clinical concern, affecting approximately 10% of pregnancies. However, only a subset of these cases reflects true pathological growth restriction [10]. Fetal growth restriction is defined by an estimated fetal weight or abdominal circumference below the 3rd centile, or an estimated fetal weight or abdominal circumference that falls below the 10th centile with any of the following: high pulsatility index of uterine artery Doppler, absent or reversed end-diastolic flow, or pulsatility index above the 95th centile of the umbilical artery Doppler [11]. The estimation of the fetal weight is not the best performer to evaluate the chances of adverse perinatal outcomes [12,13]. The fetal size is not capable of differentiating constitutionally small fetuses and pathological conditions that restrict the growth of the fetus. Therefore, close monitoring and personalized strategies for potentially high-risk pregnancies aim to reduce adverse outcomes [14].

First-trimester screening protocols have incorporated uterine artery Doppler indices, biochemical markers (pregnancy-associated plasma protein-A-PAPP-A, placental growth factor-PlGF), and maternal characteristics to predict adverse pregnancy outcomes such as preeclampsia and fetal growth restriction (FGR) [10,15,16]. There are several studies that described the role of the s-FLT/PlGF (soluble fms-like tyrosine kinase-1/placental growth factor) ratio in determining the risk of FGR and SGA fetuses [17,18]. While uterine artery resistance has been extensively studied, the role of ophthalmic artery Doppler remains less explored. Emerging evidence suggests a correlation between increased ophthalmic artery resistance and conditions associated with placental dysfunction, including preeclampsia and SGA [19,20,21].

Fetal growth restriction and small-for-gestational-age status pose significant risks for immediate and long-term outcomes. Placental insufficiency is the most frequent cause of intrauterine growth restriction [22,23]. They have prolonged hospitalization in NICU [24].

These fetuses are at high risk of stillbirth [25,26]. FGR fetuses have a higher chance of developing hypoglycemia, hypoxic–ischemic encephalopathy, gastrointestinal hemorrhage, lung bleeding, metabolic changes, or asphyxia [27,28]. They are also at high risk for future lower cognitive development as shown by a meta-analysis of Sacchi et al. [29].

The present study aims to evaluate the predictive value of ophthalmic artery Doppler parameters—specifically the peak systolic velocity (PSV) ratio and pulsatility index (PI)—for identifying pregnancies at risk of birth of SGA neonates from the first trimester.

## 2. Materials and Methods

### 2.1. Study Design and Participants:

This is a prospective observational study. We recruited 4054 women with singleton pregnancies. All these women underwent routine first-trimester screening (11^+0^–13^+6^ weeks gestation) at King’s College Hospital, London, United Kingdom. The period of recruitment for the study was between June 2019 and February 2022. The study was conducted in accordance with the Declaration of Helsinki; it was approved by the NHS Research Ethics Committee and all participants provided informed written consent.

We collected the following data: maternal age, weight, height, body mass index (BMI), obstetric history, ethnicity, smoking status, previous birth of SGA neonate, use of Aspirin, and method of conception. In order to establish the gestational age at delivery, we used the crown-to-rump length measurement (CRL) from the first trimester for all spontaneous conception pregnancies and embryo transfer date for assisted reproduction techniques (ARTs).

First-trimester assessment included measurement of mean arterial pressure (MAP), uterine artery pulsatility index (UtA-PI), pregnancy-associated plasma protein-A (PAPP-A), and ophthalmic artery Doppler indices of pulsatility index (PI) and PSV ratio. The ultrasound scans were performed by appropriately trained obstetricians and sonographers. The outcomes were divided into groups: SGA for birth weight in the ≤3rd percentile and >3rd percentile, and in the SGA with birthweight ≤10th percentile and normal birth weight (>10th percentile). We included both the 3rd and 10th centile as thresholds to distinguish between SGA and likely pathologic growth restriction. The analysis was performed in two parts. The 10th centile is used to diagnose the small-for-gestational-age fetuses that include constitutionally small fetuses and growth-restricted ones. On the contrary, according to RCOG, FGR fetuses are defined as estimated fetal weight less than the 3rd centile, which is more frequently associated with adverse perinatal outcomes [11]. By using this two-part approach, we tried to provide a better understanding of the potential role of the maternal ophthalmic artery Doppler in relationship with SGA fetuses.

We included in this observational study all women who came for a first-trimester visit at 11^+0^ to 13^+6^ weeks, and who agreed to be involved in this research. We chose to evaluate the maternal ophthalmic artery Doppler at 11 to 13 weeks + 6 days because all women are offered first-trimester screening for chromosomal abnormalities and screening for pregnancy adverse outcomes such as preeclampsia. At this moment of the pregnancy, the maternal ophthalmic artery may evidence cardiovascular changes in women at high risk for developing preeclampsia and SGA fetuses. The earliest we identify high-risk pregnancies, the better the outcome is.

The inclusion criteria were singleton pregnancies and availability of pregnancy outcomes. Pregnancies with fetal malformations were excluded. Furthermore, we removed stillbirth cases from the analysis due to the inability to evaluate the fetal weight and perinatal outcome, which are essential for a correct diagnosis.

### 2.2. Ophthalmic Artery Doppler Indices

The pregnant woman lies in a supine position. After applying a small amount of sonogel on the closed upper eyelid, a linear transducer is placed on the eye. The optic nerve is identified as a hypoechogenic line below the eyeball. The color flow Doppler identifies the ophthalmic artery superomedial to the optic nerve. The angle of insonation is less than 20 degrees, the gate is 2 mm, the wall filter is 60 Hz, and the PRF (pulse repetitive frequency) is 2–4 kHz [30].

We performed 4 measurements, two for each eye, alternatingly for each case involved in this study [31]. We recorded 3–5 waves with pulsed wave Doppler. Following the ALARA (as low as reasonably achievable) principle, the examination time was under 1 min [32]. The ophthalmic artery waveform consists of two peak systolic velocities (PSV1 and PSV2). The average of all 4 measurements was recorded [31].

### 2.3. Outome Collection

The primary outcome was to identify SGA neonates, with birthweight in the <10th percentile for their own gestational age. SGA also includes fetal growth restriction babies who are healthy [10,33]. The estimated fetal weight used charts provided by FMF Fetal and Neonatal charts [34]. SGA refers to a fetal weight below the 10th percentile for its own gestational age [10]. Fetal growth restriction is defined by an estimated fetal weight or abdominal circumference below the 3rd centile, or an estimated fetal weight or abdominal circumference that falls below the 10th centile with any of the following: high pulsatility index of uterine artery Doppler, absent or reversed end-diastolic flow, or pulsatility index above the 95th centile of the umbilical artery Doppler [32,33]. We did not differentiate fetuses with real growth restriction and small-for-gestational-age fetuses.

### 2.4. Data Analysis

Data were analyzed by using Microsoft Excel and IBM SPSS Statistics for Windows, version 30.0 (IBM Corp., Armonk, NY, USA). Kolmogorov–Smirnov and Shapiro–Wilk tests were used for normality of the data.

Continuous variables (maternal age, height, weight, BMI, PAPP-A MoM, UtA-PI MoM, MAP MoM, gestational age at delivery, birth weight) are recorded as mean ± standard deviation (SD). An independent samples t-test was performed to compare the two groups. Categorical variables (method of conception, low-dose Aspirin, smoking, ethnicity, previous history of SGA) are expressed as frequencies and percentages (*n*; %). The chi-square test (χ^2^) and Fisher’s exact test were also used for comparison between groups. Receiver Operating Characteristic (ROC) curve analysis was performed to evaluate the discriminatory ability of individual variables in differentiating between SGA with birth weight in the ≤ 3rd percentile and >3rd percentile, and also for the ones in the ≤10th percentile and >10th percentile. The area under the curve (AUC) was used as a measure of diagnostic performance. Statistical significance was relevant for a *p*-value < 0.05 for differences between groups.

## 3. Results

The study population includes 4054 pregnancies. A total of 485 (11.96%) of them had a birth weight in the ≤10th percentile and 3569 (88.03%) in the >10th percentile. Pregnancy and maternal characteristics are presented in Table 1a,b. The mean maternal age was 32.8 ± 4.83 years. Most of the cases had spontaneous conception (95%), 2.3% were smokers, and half of them were nulliparous (51.5%).

We divided the statistical analysis into two parts. First, we compared the groups of women who delivered SGA babies in the ≤3rd percentile and we compared them with the ones above it; in the second part, we compared pregnancies with SGA in the ≤10th percentile and >10th percentile.

When we compared both groups in the ≤3rd centile and ≤10th centile with >3rd centile and >10th centile, there were statistically significant differences in terms of maternal age, weight, height, BMI, PAPP-A MoM, UtA-PI MoM, gestational age at delivery, race, smoking status and previous SGA (*p* < 0.05). There was no significant difference in the MAP, method of conception, and low-dose Aspirin between the groups.

First analysis was performed by independent samples t-test for comparisons between groups. The PSV ratio in the SGA ≤ 3rd percentile, compared to SGA > 3rd percentile, and SGA ≤ 10th percentile, compared to SGA > 10th percentile was significantly higher, whereas the PI was not significantly different in either group (Table 2 and Table 3).

In both comparisons, the *p*-value for the PSV ratio was below 0.05, which is statistically significant.

**Figure 1:**
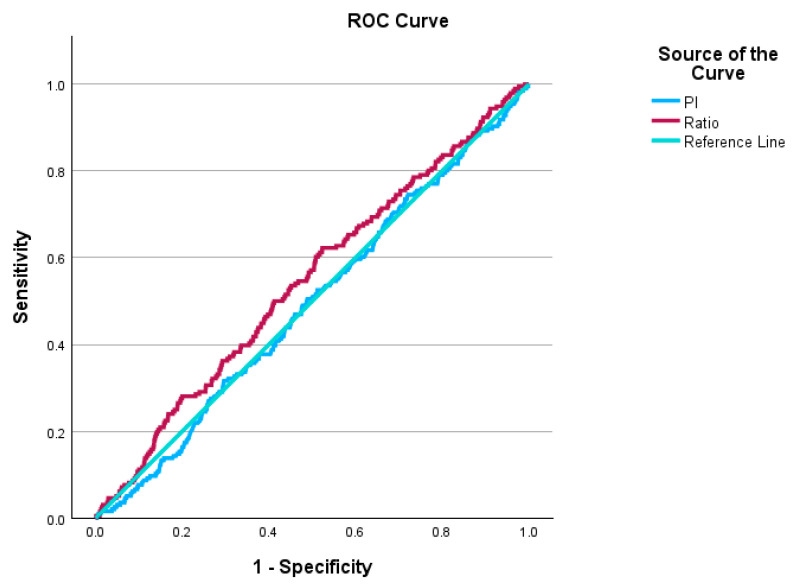
ROC curve of PSV ratio and PI for SGA below the 3rd percentile.

The area under the curve (AUC) was used as a measure of diagnostic performance of PSV ratio and PI in the SGA below and above 3rd percentile groups. (Figure 1). For PI, the AUC is 0.492, *p*-value = 0.715, 95% CI: 0.452–0.533. For the PSV ratio, the AUC is 0.544, *p*-value = 0.038, 95% CI: 0.502–0.585.

**Figure 2:**
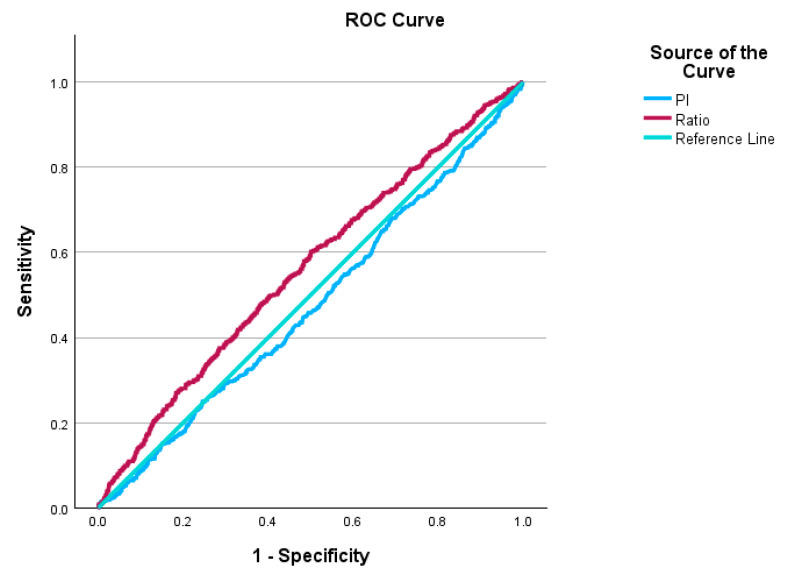
ROC curve of PSV ratio and PI for SGA below the 10th percentile.

Similarly, when we analyze the groups of SGA below and above the 10th percentile, the AUC for PI is 0.476, *p*-value = 0.088, 95% CI: 0.448–0.504. For the PSV ratio, the AUC is 0.56, *p*-value is < 0.0001, 95% CI: 0.533–0.588 (Figure 2).

## 4. Discussion

The objective of our study was to identify as early as possible in pregnancy the pregnancies that are at high risk of having a small-for-gestational-age fetus. This condition is known to have a higher incidence of adverse outcomes such as stillbirth, increased hospitalization of the newborn, and higher rates of morbidities than normally grown fetuses because of abnormal placental perfusion [35].

Our study suggests that ophthalmic artery Doppler assessment and measurement of PSV ratio in the first trimester is weak in predicting birth of SGA neonates.

Our findings show that PI does not have a discriminatory capacity to identify SGA fetuses, neither below the 3rd or 10th percentile.

The statistical analysis shows a weak correlation between the ophthalmic artery PSV ratio and prediction of SGA fetuses. Although the *p*-value is less than 0.05 when comparing the groups, the area under the curve (AUC) for both comparisons from our study is 0.54 and 0.56, respectively.

Despite these associations, it is important to note that the diagnostic performance of the ophthalmic artery PSV ratio as a standalone screening tool remains modest. In our study, the AUC for predicting SGA was below 0.6, indicating limited discriminatory power.

The slightly increased PSV ratio in SGA pregnancies is consistent with placental dysfunction, which aligns with previous studies indicating that abnormal ophthalmic Doppler indices in early pregnancy can predict poor pregnancy outcomes [36]. The observed elevated PSV ratio reflects systemic vascular changes, possibly due to impaired placental perfusion. A study by Hata et al. showed a lower PI in ophthalmic artery Doppler assessment in the third trimester, after 34 weeks, which is consistent with lower vascular resistance, even in normotensive pregnancies [37].

The ophthalmic artery Doppler provides a window into maternal vascular changes, which can be directly impacted by placental function. A previous study indicates that the best predictors of small-for-gestational-age status in the first trimester are uterine artery pulsatility index and placental growth factor [38]. Small-for-gestational-age status is a complex and heterogeneous condition that includes constitutional small fetuses and fetal growth-restricted fetuses [39]. We did not distinguish these two categories in our study.

Ophthalmic artery Doppler indices did not perform well as an individual marker in routine first-trimester screening for SGA. SGA fetuses are at heightened risk for adverse perinatal outcomes, including increased rates of morbidity and mortality. There is a high risk of stillbirth, neonatal hypoxia, neonatal death, and impaired neurodevelopment [40]. Continued research in this area could identify high-risk pregnancies requiring close monitoring.

The limitations of the study: The results may be biased by the unclear differentiation of real growth-restricted fetuses and constitutionally small-for-gestational-age infants.

The research of ophthalmic artery Doppler assessments could offer a non-invasive and accessible means of identifying pregnancies at risk in low-resource settings.

However, further large-scale, multicenter studies are necessary to validate these findings and to establish standardized measurement techniques and threshold values for clinical application.

## 5. Conclusions

In conclusion, while the ophthalmic artery PSV ratio shows potential as an adjunctive marker in the early detection of adverse perinatal outcomes, its utility could be maximized when used in conjunction with other established screening tools, only if validated in larger, multicentric studies. Continued research in this area holds promise for improving perinatal outcomes through earlier and more accurate identification of at-risk pregnancies.

## Figures and Tables

**Table 1:** **a**. Maternal characteristics for the comparison between neonates at or below 3rd centile and above 3rd centile. **b**. Maternal characteristics for the comparison between appropriate for gestational age (above 10th centile) and SGA (at or below 10th centile).

**a**				
**Characteristics**	**Total (*n* = 4054)**	**BW ≤ 3rd Percentile (*n* = 196)**	**BW > 3rd Percentile (*n* = 3858)**	***p*** **-Value**
Maternal age (years)	32.8 ± 4.83 years	31.64 ± 5.48	32.86 ± 4.8	<0.001 *
Weight (kg)	70.1 ± 15.17	66.19 ± 13.85	70.3 ± 15.21	<0.001 *
Height (cm)	165.84 ± 5.29	162.78 ± 6.79	166 ± 6.77	<0.001 *
BMI (kg/m^2^)	25.48 ± 5.29 kg/m^2^	24.98 ± 5.04	25.51 ± 5.31	0.174
PAPPA MoM	1.21 ± 0.66	1.06 ± 0.72	1.22 ± 0.66	<0.001 *
UtA-PI MoM	1.03 ± 0.31	1.13 ± 0.36	1.03 ± 0.31	0.001 *
MAP MoM	1.01 ± 0.08	1.01 ± 0.09	1.02 ± 0.08	0.137
Gestation at birth (weeks)	39.52 ± 1.83	37.69 ± 2.95	39.62 ± 1.7	<0.001 *
Conception (number)				0.47
IVF	184 (4.5%)	11 (6%)	173 (94%)	
Ovulation drugs	19 (0.5%)	0	19 (100%)	
Spontaneous	3851 (95%)	185 (4.8%)	3666 (95.2%)	
Ethnicity (number)				<0.001 *
Black	531 (13.1%)	40 (7.5%)	491 (92.5%)	
East Asian	93 (2.3%)	3 (10%)	90 (96.8%)	
Mixed	145 (3.6%)	10 (6.9%)	135 (93.1%)	
South Asian	274 (6.8%)	28 (10.2%)	246 (89.8%)	
White	3011 (74.3%)	115 (3.8%)	2896 (96.2%)	
Smoking (number)				0.032 *
Yes	92 (2.3%)	9 (9.8%)	83 (90.2%)	
No	3962 (97.3%)	187 (4.7%)	3775 (95.3%)	
Low-dose Aspirin (number)				0.44
No	3924 (96.8%)	189 (4.8%)	3735 (95.2%)	
Yes	130 (3.2%)	7 (5.4%)	123 (94.6%)	
Previous SGA (number)				<0.001 *
Multipara-no SGA	1842 (45.4%)	55 (3%)	1787 (97%)	
Multipara-SGA	126 (3.1%)	17 (13.5%)	109 (86.5%)	
Nullipara	2086 (51.5%)	124 (5.9%)	1962 (94.1%)	
* *p* < 0.05				
**b**				
**Characteristics**	**Total (*n* = 4054)**	**BW ≤ 10th Percentile (*n* = 485)**	**BW > 10th Percentile (*n* = 3569)**	***p*** **-Value**
Maternal age (years)	32.8 ± 4.83	32.2 ± 5.13	32.89 ± 4.79	0.003
Weight (kg)	70.1 ± 15.17	66.07 ± 14.44	70.65 ± 15.19	<0.001 *
Height (cm)	165.84 ± 5.29	163.28 ± 7.06	166.19 ± 6.69	<0.001 *
BMI (kg/m^2^)	25.48 ± 5.29	24.75 ± 5.01	25.58 ± 5.33	0.001 *
PAPPA MoM	1.21 ± 0.66	1.1 ± 0.7	1.23 ± 0.66	<0.001 *
UtA-PI MoM	1.03 ± 0.31	1.09 ± 034	1.02 ± 0.31	<0.001 *
MAP MoM	1.01 ± 0.08	1.02 ± 0.09	1.02 ± 0.08	0.523
Gestation at birth (weeks)	39.52 ± 1.83	38.57 ± 2.4	39.66 ± 1.7	<0.001 *
Conception				0.773
IVF	184 (4.5%)	25 (13.6%)	159 (86.4%)	
Ovulation drugs	19 (0.5%)	2 (10.5%)	17 (89.5%)	
Spontaneous	3851 (95%)	458 (11.9%)	3393 (88.1%)	
Ethnicity (number)				<0.001 *
Black	531 (13.1%)	84 (15.8%)	447 (84.2%)	
East Asian	93 (2.3%)	10 (10.8%)	83 (89.2%)	
Mixed	145 (3.6%)	19 (13.1%)	126 (86.8%)	
South Asian	274 (6.8%)	70 (25.5%)	204 (74.5%)	
White	3011 (74.3%)	302 (10%)	2709 (90%)	
Smoking (number)				0.003 *
Yes	92 (2.3%)	21 (22.8%)	71 (77.2%)	
No	3962 (97.3%)	464 (11.7%)	3498 (88.3%)	
Low-dose Aspirin (number)				0.14
No	3924 (96.8%)	465 (11.9%)	3459 (88.1%)	
Yes	130 (3.2%)	20 (15.4%)	110 (84.6%)	
Previous SGA (number)				<0.001 *
Multipara-no SGA	1842 (45.4%)	152 (8.3%)	1690 (91.7%)	
Multipara-SGA	126 (3.1%)	37 (29.4%)	89 (70.6%)	
Nullipara	2086 (51.5%)	296 (14.2%)	1790 (95.8%)	

BW—birth weight; SGA—small for gestational age; BMI—body mass index, UtA-PI—uterine artery pulsatility index; MoM—multiples of median; IVF—in vitro fertilization; PAPPA—pregnancy-associated plasma protein-A; MAP—mean arterial pressure; * *p*-values < 0.05 were considered statistically significant.

**Table 2:** Mean value of maternal ophthalmic Doppler indices (PSV ratio and PI) in the comparison between neonates at or below 3rd centile and above 3rd centile.

Group	BW ≤ 3rd Centile (*n* = 196)	BW > 3rd Centile (*n* = 3858)	*p*-Value
PI (mean ± SD)	1.86 ± 0.37	1.88 ± 1.39	0.475
PSV ratio (mean ± SD)	0.65 ± 0.10	0.63 ± 0.10	0.038 *

BW—birth weight; PI—pulsatility index; PSV—peak systolic velocity; SD—standard deviation. * *p* < 0.05.

**Table 3:** Mean value of maternal ophthalmic Doppler indices (PSV ratio and PI) in the comparison between appropriate for gestational age (above 10th centile) and SGA (at or below 10th centile).

Group	BW ≤ 10th Percentile (*n* = 485)	BW > 10th Percentile (*n* = 3569)	*p*-Value
PI (mean ± SD)	1.85 ± 0.40	1.88 ± 0.39	0.113
PSV ratio (mean ± SD)	0.65 ± 0.10	0.63 ± 0.10	<0.001 *

BW—birth weight; PI—pulsatility index; PSV—Peak systolic velocity; SD—standard deviation. * *p* < 0.05.

## Data Availability

Data will be provided upon request.

## Abbreviations

The following abbreviations are used in this manuscript:

AUC	Area under the curve
BMI	Body mass index
CI	Confidence interval
FMF	Fetal Medicine Foundation
IVF	In vitro fertilization
MAP	Mean arterial pressure
MOM	Multiples of median
PAPP-A	Pregnancy-associated plasma protein-A
PE	Preeclampsia
PI	Pulsatility index
PlGF	Placental growth factor
PSV	Peak systolic velocity
ROC	Receiver operating characteristic curve
SD	Standard deviation
SGA	Small for gestational age
Ut-A-PI	Uterine artery pulsatility index
